# Dual-Illumination Ultrasound/ Photoacoustic System for Cervical Cancer imaging

**DOI:** 10.1109/jphot.2020.3043685

**Published:** 2020-12-14

**Authors:** Maryam Basij, Andrei Karpiouk, Ira Winer, Stanislav Emelianov, Mohammad Mehrmohammadi

**Affiliations:** 1 Department of Biomedical Engineering, Wayne State University, Detroit, MI, USA; 2 School of Electrical and Computer Engineering, Georgia Institute of Technology, Atlanta, GA, USA; 3 Department of Oncology, Wayne State University, Detroit, MI, USA; 4 Barbara Ann Karmanos Cancer Institute, Detroit, MI, USA; 5 Department of Obstetrics and Gynecology, Wayne State University, Detroit, MI, USA

**Keywords:** Endoscope, Dual illumination, side-firing, fiber optic, Monte-Carlo, ultrasound, photoacoustic

## Abstract

Early stage cancer detection technologies can provide functional information and potentially decrease the mortality rate caused by cervical cancer. In our previous work, a miniaturized ultrasound and photoacoustic endoscopic system has been developed to image the cervical tissue through the cervical canal to fulfills the need for a safe, low-cost, and high-resolution functional diagnostic system. However, the miniaturized size of endoscope and American National Standards Institute safety limits cause constraints of using high-intensity illumination during imaging. In addition, the strong light scattering of tissues limits the light penetration depth. Fortunately, the cervix anatomy allows for the delivery of additional light from the ectocervix by using an external illumination system. Here we propose a dual, co-planar illumination system, which can provide adequate illumination to the cervical tissue via combined internal and external light delivery strategies. Therefore, an increase in the area of light-tissue interaction allows us to raise the laser light energy while keeping fluence under safety limits. Thus, a reliable PA imaging can be obtained for the whole cervical tissue thickness. The system performance was tested using a Monte Carlo simulation, and laser-light fluence was calculated and compared at different depths within a simulated cervical-tissue model. The results indicated a higher and more uniform fluence in the Monte Carlo simulations. In addition, the photoacoustic imaging of the proposed system was evaluated by two cervical tissue-mimicking phantoms with human blood and graphite rods as inclusions inside it. In accordance with the simulations, the phantom study revealed a more reliable photoacoustic signal for the entire depth of the phantoms with an improved contrast to noise ratio and signal to noise ratio, and a higher coverage ratio of the imaging field of view. In summary, the dual-mode illumination system can provide more realistic information of inclusions within the tissue while considering safety limits, which can lead to more accuracy in biomarker detection for cervical cancer diagnostics.

## 1. Introduction

According to the American cancer society report, around 1.8 million new patients will be diagnosed with cancer in the United States in 2020 [1]. Cervical cancer with the fourth-most common cancer and the second leading death cause of cancer-related among young women would be considered as one of the serious concerns nowadays [2, 3]. Currently, cervical cancer is diagnosed through routine screenings or pelvic examinations. Although for cancer screening and diagnosis the cervix and vaginal mucosa is visualized by speculum examination and evaluated with pap smear, the cervix might appear normal when the disease is microinvasive or inside the endocervical canal [4]. Therefore, alternative detection measures will hopefully enable early diagnosis of pre-cancerous lesions and microscopic disease, hence reducing both morbidity and mortality [5, 6]. In addition, functional information regarding tumor localization can conceivably improve treatment planning. For example, additional information could be used to determine if the patient is a candidate for surgery or radiation therapy [7, 8]. The existing imaging modalities, including ultrasound (US), magnetic resonance imaging (MRI), and computed tomography, are limited by key factors, including cost-effectiveness, compactness/point-of-care, and accuracy. These unmet clinical needs call for the development of a simple, accurate diagnostic imaging system which can acquire high-resolution images of regions in close proximity to the cervical tissue in the point of care setting.

Photoacoustic (PA) imaging is a hybrid imaging modality that extracts the optical properties of the tissue of interest by acquiring its thermoelastic responses upon irradiation with short (in the order of nanoseconds) laser pulses. PA imaging can be easily integrated with a US imaging system because the same US transducer can be used for acquiring the PA signals, and minimal modification is required in the imaging sequence as US/ PA images are naturally co-registered. PA imaging can also provide spatial distribution of the tissue’s optical absorption components [9–12]. The dependence of the PA signal on the optical absorption properties can be exploited to distinguish between different chromophores, including melanin, hemoglobin, fat occurring within a tissue or organ. It has been established from a plethora of previous studies that increased vascularity within cervical cancers is related to tumor malignancy [13, 14]. Accordingly, hemoglobin can be used as an endogenous contrast agent for PA imaging [15]. In addition, several studies have utilized spectroscopic PA (SPA) for measuring blood oxygenation and tissue hypoxia [16–19]. Therefore, treatment planning can be improved by using SPA imaging and providing the hypoxia map of cervical tissue. Overall, PA imaging can play an important role as a non-invasive, safe, real-time, and economical diagnostic tool for detecting both pre-cancerous and cancerous lesions and ultimately assisting in the development of treatments for cervical cancer patients.

**Figure 1 :**
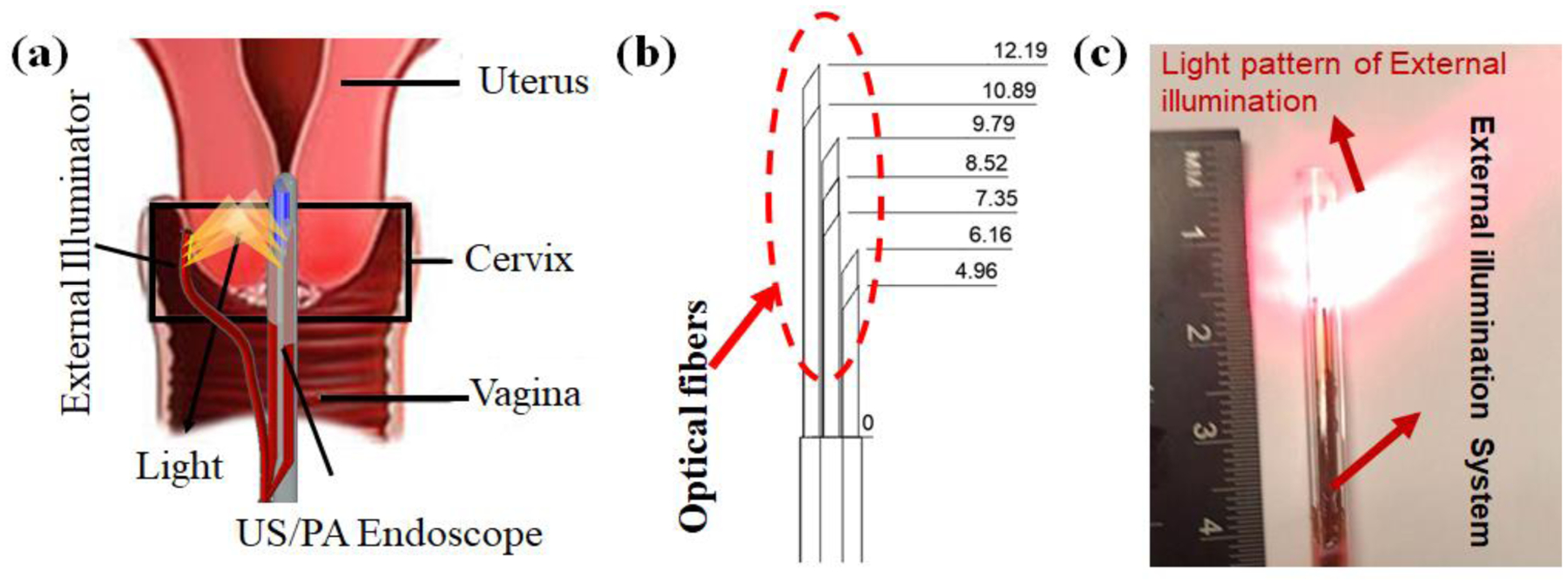
A schematic of the dual-illumination US/PA endoscope-based imaging system for imaging the cervical tissue. When the integrated endoscopic imaging system passes through the cervical canal, the external light delivery system provides illumination through the ectocervix. (b) A schematic of our external illumination fiber equipped with seven optical fibers. The arrangement of the fibers is shown with their distance to the reference point on fiber bundle. (c) An image of the external light delivery system and its light pattern.

In our previous work, a miniaturized US/PA imaging system was able to provide concurrent structural, functional, and molecular information about the tissue of interest [20–23]. In the US/PA endoscopic system, the laser light was delivered to the tissue using a compact optical fiber-based system. The small size of the combined probe (with a diameter of 7.5 mm) allows it to be placed close to the cervical and endocervical tissue. The endoscope has the ability to provide high-resolution US/PA images. In this case, the light delivery was limited to six optical fibers for the purpose of keeping the probe size to a minimum. Therefore, the limited pulsed laser light can be carried to the tissue. In addition, the American National Standards Institute (ANSI) safety restrictions [24, 25] limit the amount of fluence provided by our light delivery system, which reduces the imaging depth of the system. On the other hand, the anatomical structure of the cervical tissue supports the need for developing an external light delivery system along with the internal light delivery equipped endoscopic system. Hence, our proposed system will uniformly illuminate the tissue, maintaining a low fluence, and produce comparable results independent of imaging depth (Fig 1.a).

In this study, we introduce a dual-illumination US/PA imaging system for high fidelity imaging of the cervical tissue, by irradiating it from both the endocervical canal and ectocervix. The external system, with a diameter of 4.2 mm, consists of seven optical fibers that deliver the illumination. The propagation of light within the tissue, provided by different illumination strategies, was simulated using the Monte Carlo method and the PA imaging performance was evaluated using cervical tissue mimicking phantoms.

## 2. MATERIALS AND METHODS

### 2.1. Design of a co-planar, combined internal and external light delivery system

The dual-mode illumination endoscopic system consists of two components: 1) an integrated US/PA, internal light delivery equipped endoscopic system, 2) an external light delivery system. The internal component of the endoscopic system consists of a phased-array ultrasound probe coupled with six polished optical fibers. The total diameter of the integrated US/PA imaging system is approximately 7.5 mm. The design of an external illumination system for our endoscopic device required a side-firing light delivery technique able to illuminate the same region of tissue as the integrated US/PA imaging system. Therefore, the external illumination system was developed using seven core/cladding silica fibers (FIP600660710, Molex Inc., USA). The fibers were polished at an angle of 38° to guide the light and irradiate the sides of the tissue. To ensure adequate tissue illumination, the fibers were placed in a specific arrangement, as shown in Fig 1.b. The total diameter of the external fiber measures 4.2 mm. In accordance with the total reflection approach, an increase in the delivered light beam was achieved by housing the fibers within a borosilicate tube and maintaining the air/silica interface for total internal reflection (TIR) [26]. Furthermore, the borosilicate tube adds a layer of protection for the fragile polished fibers. Fig 1.c illustrates the light pattern of the proposed external light delivery system.

### 2.2. US and PA Data Acquisition System

**Figure 2:**
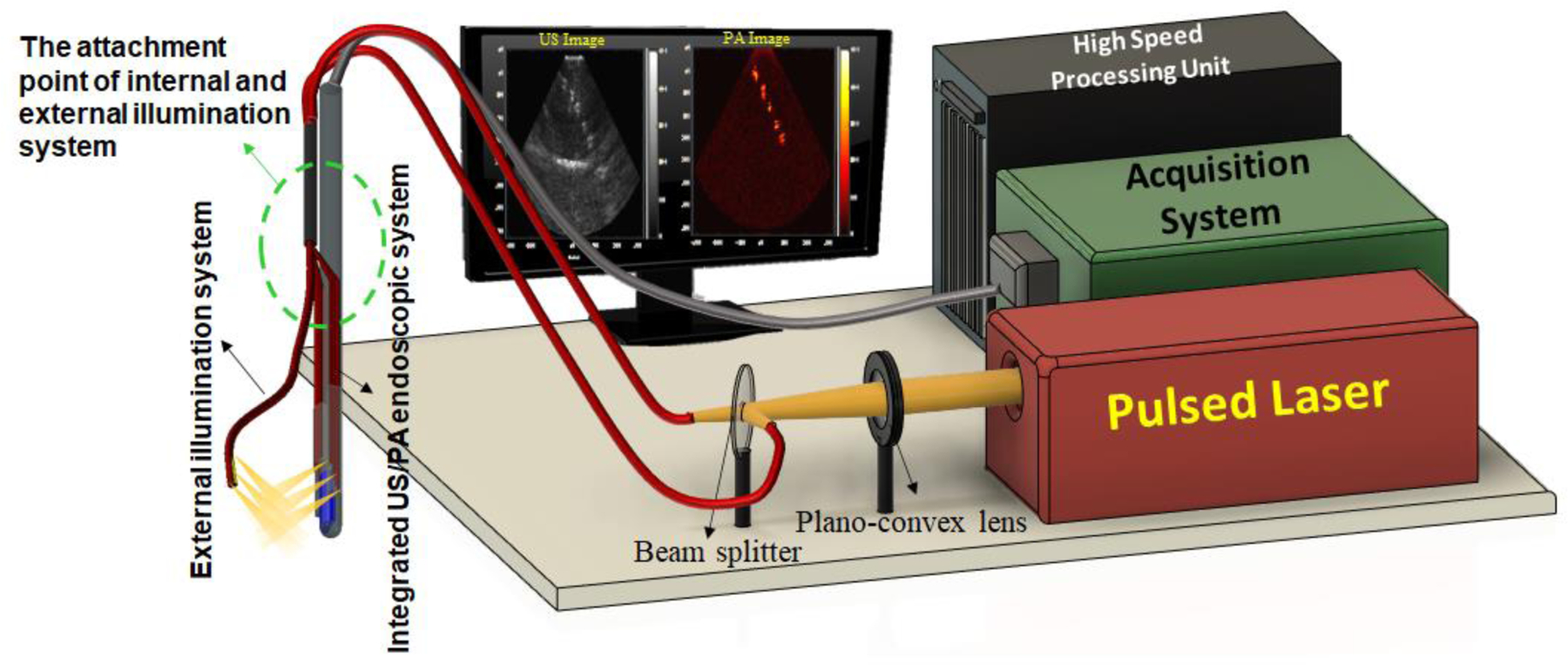
A schematic of the imaging system components for the dual-mode illumination US/PA endoscopic system. 680 nm pulse laser is used for generating PA images. A plano-convex lens is utilized for focusing the laser beam, and a beam splitter couples the pulsed laser light into the internal and external fiber bundles. The internal and external fibers are attached to each other before the distal end to ensure that the external light beam in positioned in front of the integrated US/PA endoscopic system.

Fig 2 diagrams the overall architecture of the dual-mode illumination US/PA imaging system. For photoacoustic imaging, a tunable pulsed laser (Phocus Core, OPOTEK® Inc., USA) was used, operating at a wavelength of 680 nm and a repetition rate of 10Hz. The output laser beam is focused by a plano-convex lens (LA1134-A, Thorlabs Inc., USA) to be the same size as proximal ends of bundled fibers. A beam splitter (EBS1, Thorlabs Inc., USA) was utilized to couple the laser beam into both the internal and external light delivery systems. To retain the external fiber bundle in front of the US probe active aperture consistently, the external and internal light delivery systems are bundled together at a distance of about 100 mm near its distal end (green dashed line in Fig 2). For acquiring US/PA images, the endoscope was connected to a programmable US engine (Vantage 128, Verasonics Inc., USA) through an in-house designed adaptor.

### 2.3. Monte Carlo simulation study

**Figure 3:**
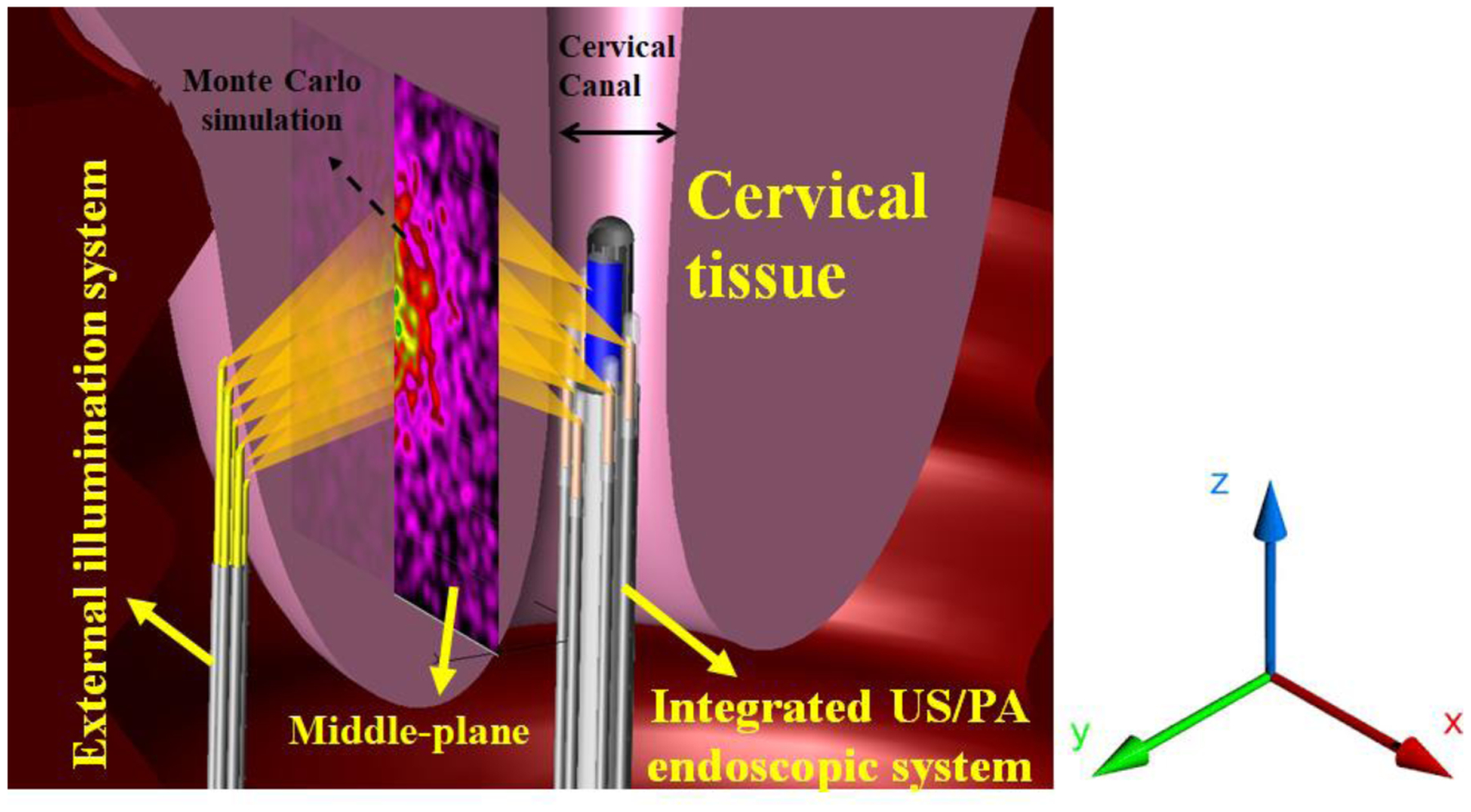
A schematic of 3D design used for the Monte Carlo simulation of the dual-illumination imaging system. A cervical tissue with 30 mm thickness was irradiated by the external and internal illumination systems. A perfect absorber plane was placed at different depths to measure the light ray fluence.

The light propagation inside the cervical tissue is simulated with different illumination strategies by a Monte Carlo modeling software (TracePro Software, Lambda Research Corporation, MA, USA), and the fluence was calculated at different depths. In the simulations, a large number of photons are propagated and tracked inside the tissue based on the probability of the tissue reflection, refraction, absorption, and scattering characterizations [27]. For the simulations, a 3D model for the external and internal light delivery systems was defined, and the geometry of 30 mm thick cervix tissue was utilized for analyzing the propagation of light (Fig 3). The optical properties of tissue, including anisotropy of 0.9 g, scattering coefficient of 0.72 1/mm, the absorption coefficient of 0.082 1/mm, and optical index of 1.44, as derived from previous studies [28, 29]. Since the endoscope and the external illumination device are envisioned to be attached to the cervical tissue, a cubic homogenous volume of the tissue was considered in our simulation studies, and the tissue curvatures were ignored. The tissue response was obtained at different depths at a wavelength of λ =680 nm with 1.5 and 1.75 m-Watts for internal and external illumination systems, respectively.

### 2.4. Evaluating the effectiveness of the dual-mode illumination system

**Figure 4:**
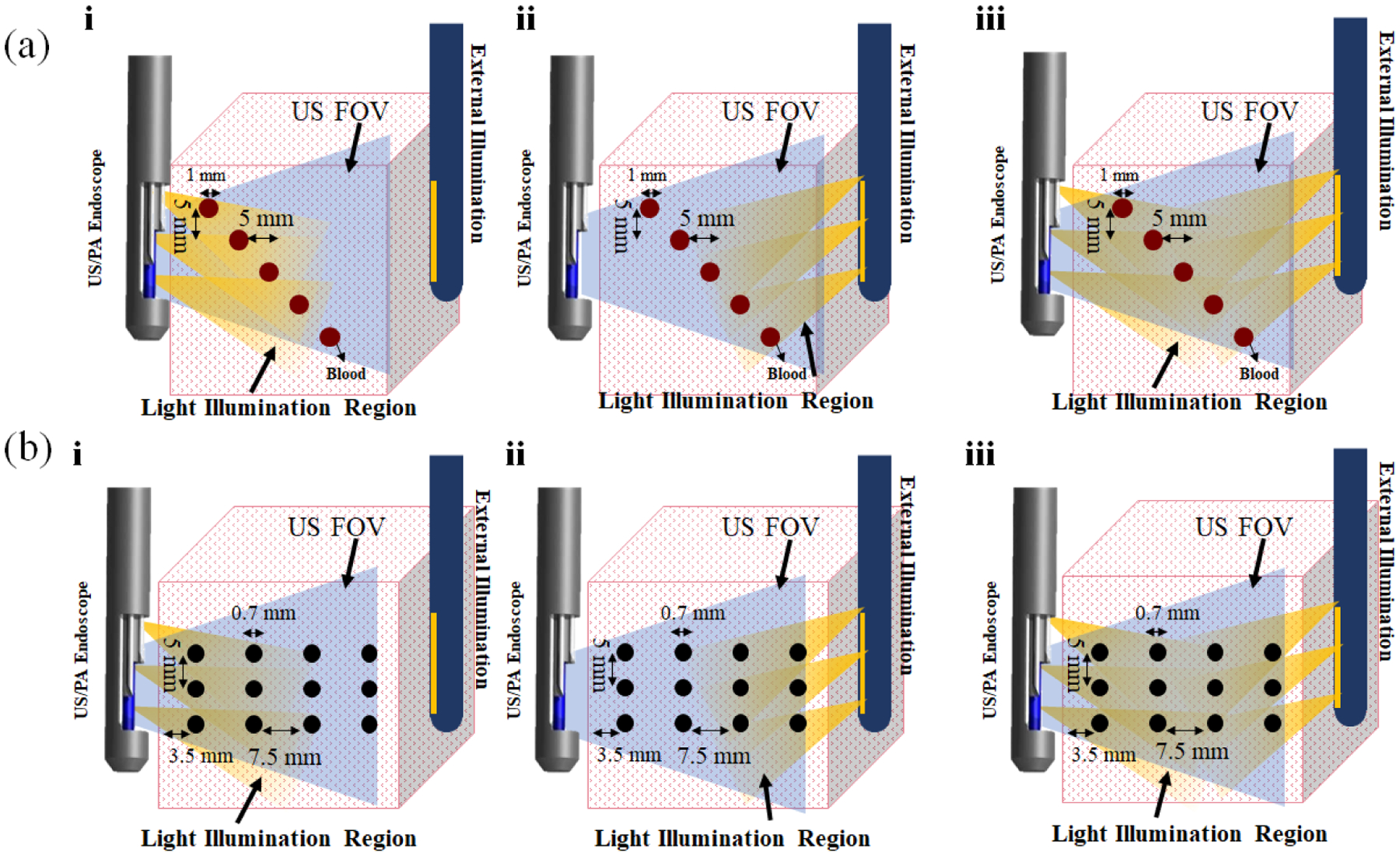
A schematic of cervix mimicking phantoms made using porcine tissue embedded (a) with five US-transparent tubes filled with human blood with an interval distance of 5 mm (b) with twelve graphite rods with vertical and horizontal interval distance of 5 mm and 7.5 mm. The phantoms were imaged by applying three illumination techniques: (i) internal, (ii) external, and (iii) combined dual-mode illumination. The light pattern and US field of view are indicated by yellow and blue color, respectively.

The efficacy of the dual-mode illumination system was evaluated using two 30 mm thick porcine tissue-cervical mimicking phantoms. The thickness of the phantom was selected to match the average thickness of the human cervix tissue. The two phantoms were used to measure and compare the performance and the tissue coverage ratio of the internal and external illumination systems. The five polytetrafluoroethylene (PTFE) tubes with a diameter of 1 mm ( ZEUS Inc., USA) filled with heparinized human blood were placed at different depths at 5 mm increments inside the first porcine tissue phantom (Fig 4.a). The second phantom was made of a grid of twelve graphite rods (diameter: 0.7 mm) placed within the tissue at 5 mm and 7.5 mm distance intervals in the vertical and horizontal axes, respectively (Fig 4.b). The combined internal and external illumination was evaluated by analyzing the PA data acquired with the 1) internal light delivery equipped US/PA endoscope, 2) external light delivery system, and 3) combined internal and external light delivery system. PA images were acquired at the average laser fluence of 2.76 and 11.19 mJ/cm^2^ for internal and external light delivery systems, respectively, at the λ = 680 nm wavelength. The light fluence from the nearest fibers to the tissue that represents the maximum fluence on tissues was calculated as 25.07 and 20.50 mJ/cm^2^ for an integrated system and external illumination system, which is below the maximum permissible exposure (MPE) safety limit (30 mJ/cm^2^ for 680 nm). The integrated endoscope systems were aligned by imaging a mesh-grid calibration phantom prior to the experiments. The US/ PA signals were acquired by the programmable data acquisition system.

## 3. RESULTS AND DISCUSSION

### 3.1 Comparison of light fluence at different depths using Monte Carlo simulation

**Figure 5:**
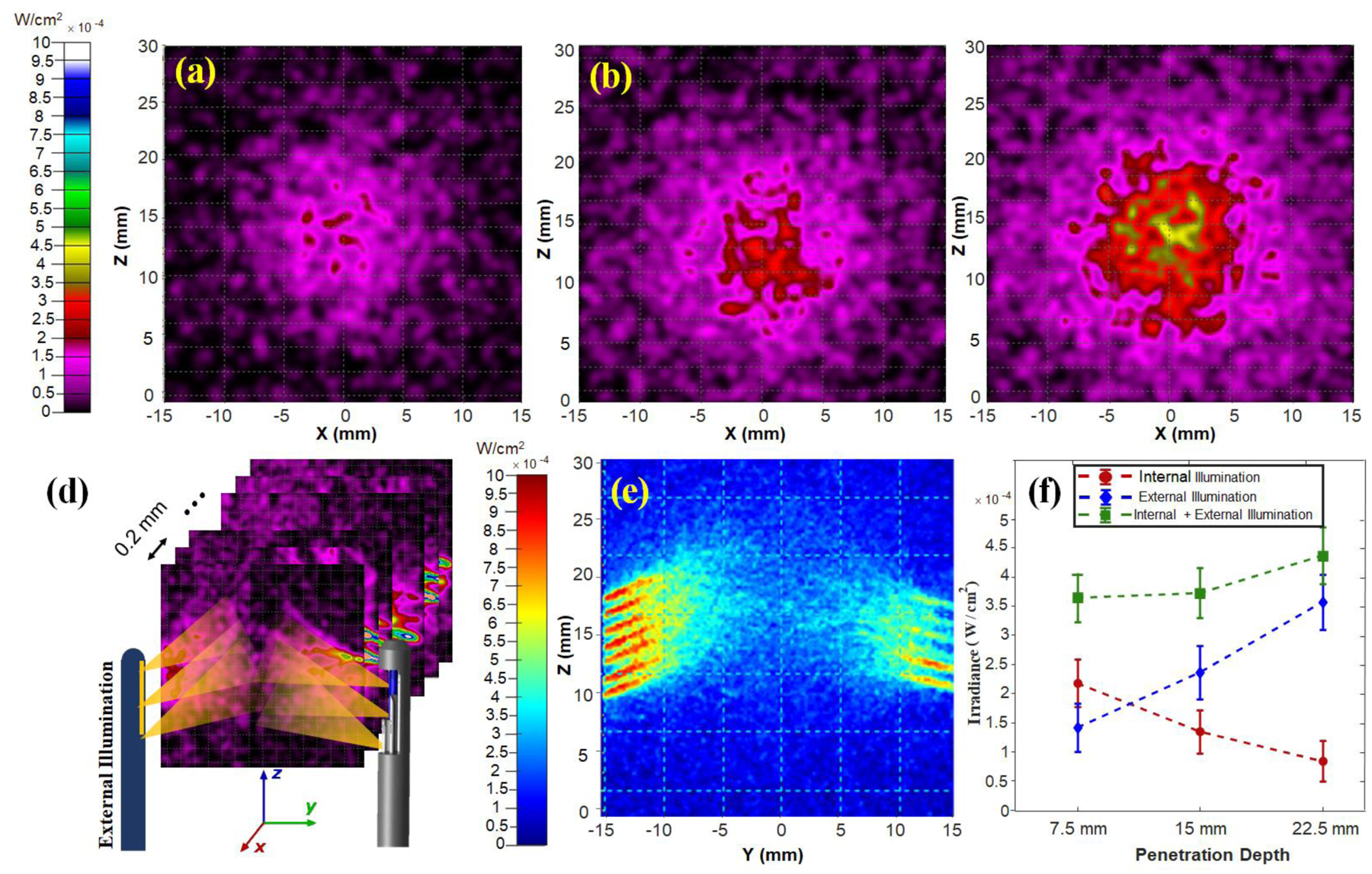
Monte Carlo simulation of the light fluence for each illumination approach inside the cervical tissue with a thickness of 30 mm. (a-c) Simulated middle plane at 15 mm of each illumination strategy (d) Simulation of light pattern inside the tissue on fourteen sagittal planes with the increments of 0.2 mm along X-axis (e)The obtained light patten of internal and external illumination system on YZ plane by averaging the simulated light pattern over fourteen planes covering the elevational beam width of the US transducer (f) The mean and standard deviation of the incident light ray fluence of planes at depths of 7.5,15 and 22.5 mm from integrated US/PA endoscope. The results show how a higher and more consistent fluence can be obtained throughout the whole simulated cervical tissue thickness by using the dual-illumination system.

The light fluence for each illumination strategy was evaluated by placing a perfect absorber sheet inside the simulated cervical tissue, and ray fluence was calculated at different depths using the Monte Carlo method. The absorber sheet with dimensions of 40×40 mm and resolution of 1024×1024 was utilized for the simulation. Fig 5.a–c shows the results from the middle plane inside the tissue when it has equal distance (15 mm) from both the internal and external illumination systems. The results indicated a higher light fluence and greater coverage area for the combined modality system. The results of the light diffusion simulation, over fourteen sagittal planes with increments of 0.2 mm along the X-axis (Fig 5.d) and the averaged light patten of dual illumination system on YZ plane inside the tissue, as shown in Fig 5.e. These fourteen sagittal planes cover a thickness of 2.8 mm slice, which corresponds to the elevational beam width of the endoscopic US transducer.

Fig 5.f shows the mean and standard deviation of irradiance for incident rays of perfect absorber sheets at 7.5, 15, and 22.5 mm depths from the tissue surface on the cervical canal in XZ plane. It clearly demonstrates that the higher light energy to the imaging depth can be achieved using the dual-illumination method. In fact, the simulation results demonstrate the superior capabilities of the combined external and internal illumination to provide uniform illumination throughout the tissue thickness and, therefore, to reveal all the absorbing areas independently depth. In addition, a higher light fluence in deeper tissue could lead to an improvement of PA signal-to-noise ratio.

### 3.2 Evaluating the efficacy of the dual-mode illumination system in tissue-mimicking phantoms

**Figure 6:**
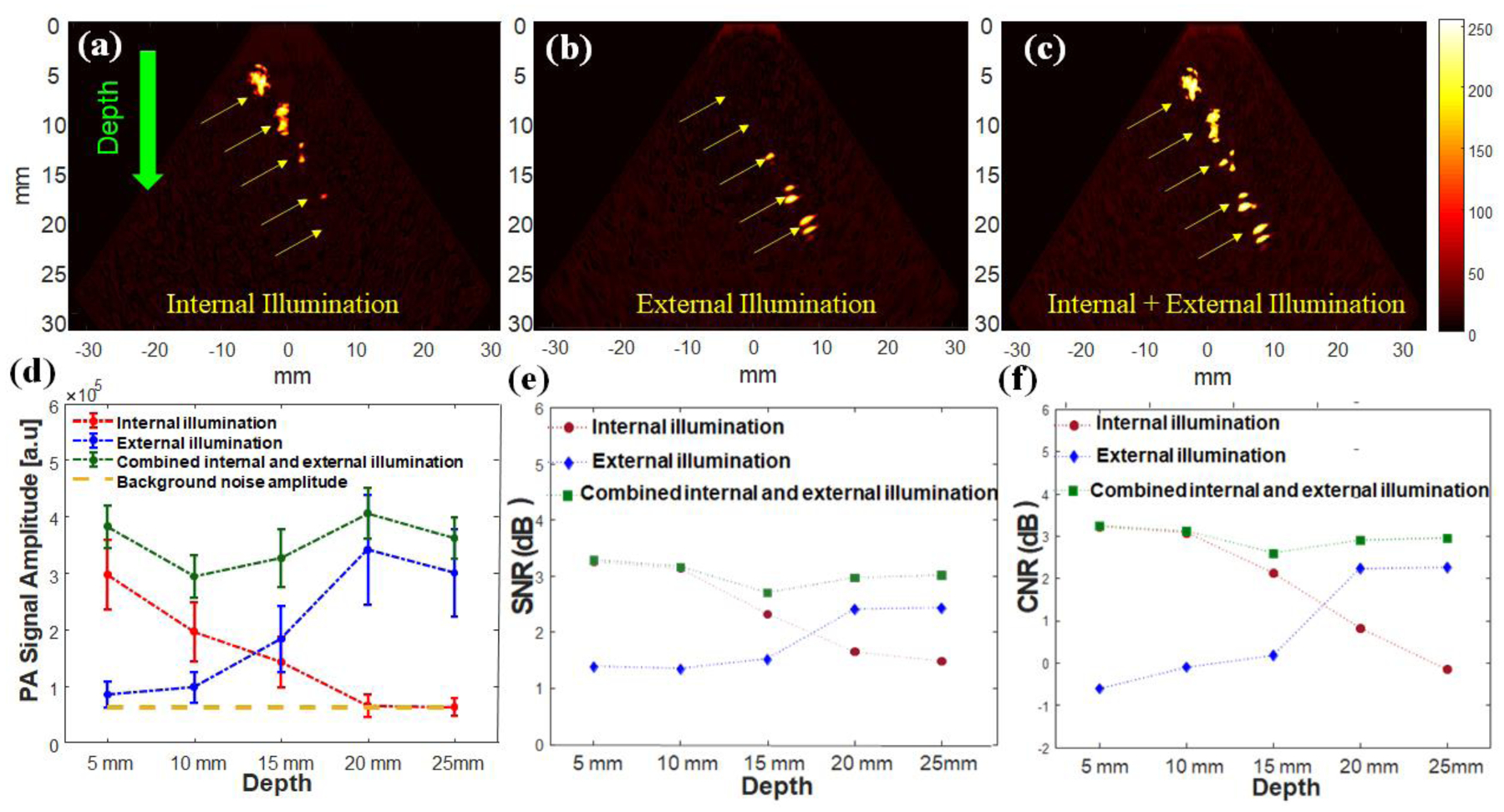
PA images of cervix mimicking tissue phantoms containing five blood filled tubes illuminated using three strategies: (a) internal, (b) external, and (c) dual-mode illumination. (d) PA amplitude, (e) the SNR and (f) the CNR of blood-filled tubes at different depths for each illumination method demonstrates the uniformity of the PA signal across the tissue with the combined dual-mode illumination system.

Fig 6.a–c shows the acquired PA images of the tissue-mimicking phantom containing blood-filled tubes illuminated using the described methods. Light scattering and attenuation inside the tissue affect the PA signal intensity when imaging depth increases for either the internal or external illumination systems. On the contrary, for the combined illumination system, all the inclusions within the phantom can be easily distinguished and have strong PA signals throughout the whole phantom thickness. The PA images of the phantom studies were quantitatively analyzed by computing the PA signal amplitude, the signal-to-noise ratio (SNR), and the contrast-to-noise ratio (CNR) of each inclusion (Fig 6.d e and f, respectively). The PA amplitude of the signal from each inclusion was computed by averaging the signals within a manually defined region of interest (ROI). The circular-shape regions with a diameter of 1 mm (size of inclusions) were selected individually, and an averaging was applied in each area. The surrounding region in the sector-shape PA image was utilized as the background region. In conjunction, Fig 6.d demonstrates that the PA amplitude is uniform for all inclusions when the dual illumination system is used because it provides a uniform light influence inside the tissue. The depth independency of the PA signal in the combined illumination method is desirable for imaging devices since it can eliminate any misinterpretation of varied signal amplitudes appearing at different imaging depths. For instance, a PA signal from a large blood vessel deeply located in the tissue can have the same amplitude as the small one located in a shallow depth when illuminated using either the internal or external illumination strategies. In addition, small inclusions located deep within the tissue can be eliminated or can be perceived as the background noise, similar to the inclusions located at depths higher than 15 mm in imaging with an internal illumination system (red dashed lines in Fig 6.d). The results in Fig 6.e–f indicates that the dual-mode illumination system can provide higher, more consistent SNR and CNR over the entire phantom thickness. The light fluence of the dual-mode illumination system complies with ANSI safety limits. The SNR and CNR results emphasize how using combined illumination can provide a similar contrast between objects and background and create a real spatial distribution of components inside the tissue.

**Figure 7:**
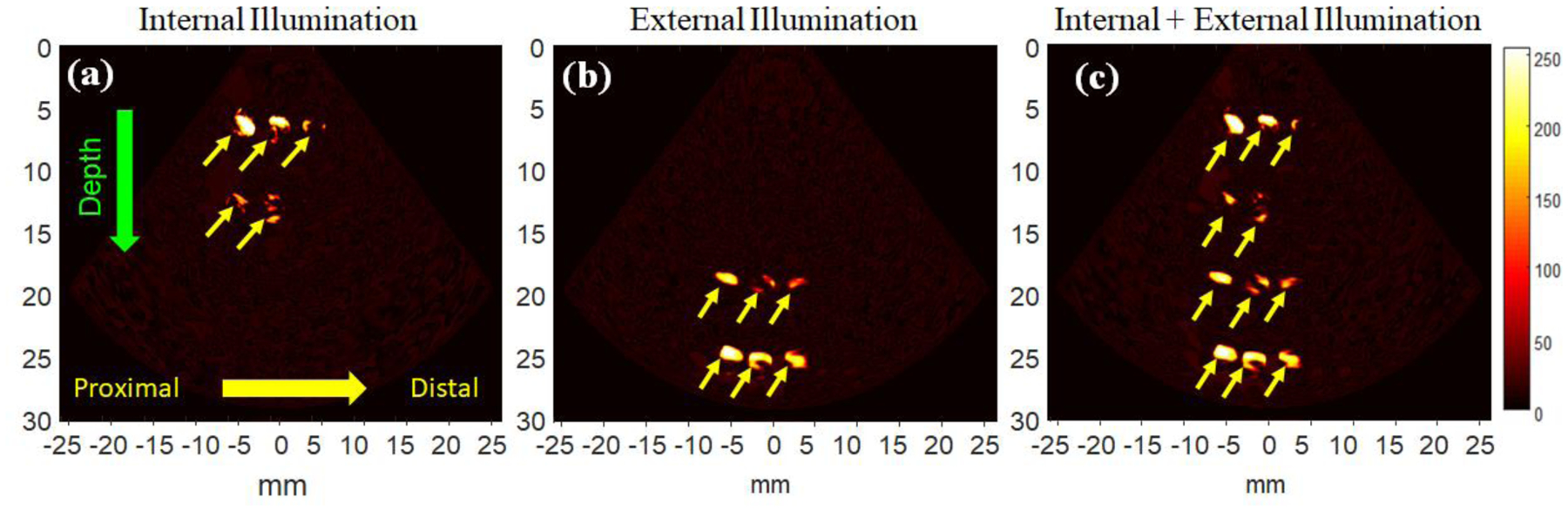
PA images of cervix mimicking tissue phantom containing twelve graphite rods illuminated using (a) internal, (b) external, and (c) combined dual-mode illumination systems. The results indicated that about 41%, 48%, and 91% of coverage percentage of tissue can be obtained from internal, external, and dual-illumination systems, respectively.

Furthermore, the coverage of the cervix mimicking tissue was calculated as the ratio of the area covered by PA imaging for each illumination approach and represented as a coverage percentage (%). PA data was acquired by imaging the phantom containing graphite rods. The inclusion’s pattern inside the tissue was considered as a grid to evaluate the coverage area of the illumination systems. Therefore, the coverage area was extracted by applying an adaptive thresholding method, such as the Otsu algorithm [30], on the PA signal amplitude to remove the environment background noise. The Otsu’s method is an automatic image thresholding algorithm that separates pixels into two classes of foreground and background based on pixels’ intensity variance. Hence, the coverage area of each inclusion region was selected manually as previously, and the ratio of detected pixels to total expected pixels inside the image was calculated as the coverage percentage. Fig 7 shows the improvement of the coverage ratio using the dual-illumination system in whole phantom thickness. The results reveal that approximately 41%, 48%, and 91% of the coverage area is obtained for internal, external, and dual-illumination systems. As shown in Fig 7.c, one of the inclusions located at a depth of 15 mm could not be visualized using the dual-mode illumination system, which can be explained by the light delivered from the internal illumination system. As mentioned above [20–22], the internal illumination system consists of six fibers: two on top and four attached to the sides of the US probe. Although the light illumination with full coverage is provided for the endoscope’s field of view by the pre-designed arrangement of side fibers, the fibers on the top and close to the proximal end of the US aperture deliver a larger fluence of light since the light coming out of these fibers have a shorter travel path before it enters the tissue. Therefore, as demonstrated in Fig 7.c, the objects which are closer to the proximal end of the probe generated a higher PA signal, and the objects closer to the distal end and deeper within the tissue can potentially be missed. This issue can be resolved by increasing the light input of the fibers placed on the side of the probe or by utilizing a weighting matrix on the acquired PA images to compensate for fluence inhomogeneities. The weighting matrix can be achieved accurately by the Monte Carlo simulation. While proposed dual illumination method can enhance the coverage and penetration depth of PA imaging, it is limited by the anatomical features of the tissue to allow for the placement and movement of the external and internal illumination in a co-planar arrangement. For the case of cervical tissue, such arrangement is doable due to the possibility to have light illumination within the cervical canal and on external surface of the cervix within the vagina.

## 4. Conclusions

Our previous papers have introduced a miniaturized US/PA imaging system for cervical cancer visualization. However, the ANSI safety standards limit light fluence on the sample’s surface, and the proposed light delivery system containing the six optical fibers cannot deliver sufficient light to image the entire depth of the tissue due to strong light scattering inside the cervical tissue. The anatomy of cervix tissue provides an opportunity to develop an additional light delivery system from the vaginal fornices. In this study, an external light delivery system was introduced and integrated with our endoscopic system. The dual-mode illumination system provides sufficient illumination to the cervical tissue from both the cervical canal and the ectocervix. The external light delivery system was developed using seven optical fibers, which were sealed with a borosilicate tube. The total diameter of the external fibers equals to 4.2 mm. A Monte Carlo simulation was utilized to compare the fluence delivered to tissue at different tissue depths. The simulation results demonstrated that a higher and more consistent uniform light fluence could be obtained through the dual-mode illumination system.

Further, the PA images of the two cervix mimicking tissue phantoms were quantitatively analyzed to compare the performance of internal, external, and dual-mode illumination systems. The results demonstrated that neither the internal nor the external illumination system operated individually could reliably cover the entire depth of the cervix if the laser fluence is kept under the safe level. In addition, an improved CNR and SNR for the dual-mode illumination system verified the system performance and imaging validation. Moreover, the phantom studies revealed that the dual-mode illumination system provided higher tissue coverage.

In summary, integrating the external light delivery system with a US/PA combined endoscopic system demonstrated an improvement in the PA imaging accuracy and coverage area while remaining within the safety limitations of the system. The dual-mode illumination system can identify an increase in cervical cancer biomarker detection since it provides a more reliable PA signal and decreases imaging depth dependency. In addition, an improvement in treatment planning can be obtained by reaching to the functional information regarding tumor localization. Further research is required to evaluate the system performance on human cervical tissue.
